# Supplementary material for: Inhibitory muscarinic acetylcholine receptors enhance aversive olfactory learning in adult Drosophila
Source: eLife. 2019 Jun 19;8:e48264. doi: 10.7554/eLife.48264 (PMC6641838; doi:10.7554/eLife.48264)
Supplement: Supplementary file 1. [file elife-48264-supp1.docx]

**Table S1: Statistical analysis­**

| **Figure** | **Data** | **Statistical method** | **Comparison** | **P-value/ adjusted P-value** | **Significance** |
| --- | --- | --- | --- | --- | --- |
| Figure 1A | Real time PCR of RNAi 1 (TRiP) | One way non parametric ANOVA -  Kruskal-Wallis test | ANOVA   1. ELAV-GAL4 2. UAS-mACR-A-RNAi-1 3. ELAV-GAL4>UAS-mACR-A-RNAi-1 | < 0.01 | ** |
|  |  | Dunn's multiple comparisons test | 1. ELAV-GAL4 2. UAS-mACR-A-RNAi-1 | >0.9999 | n.s. |
|  |  |  | 1. ELAV-GAL4 2. ELAV-GAL4>UAS-mACR-A-RNAi-1 | 0.0117 | * |
|  |  |  | 1. UAS-mACR-A-RNAi-1 2. ELAV-GAL4>UAS-mACR-A-RNAi-1 | 0.0227 | * |
|  | Real time PCR of RNAi 2 (VDRC) | Welch ANOVA | ANOVA   1. ELAV-GAL4 2. UAS-mACR-A-RNAi-2, UAS-DCR 3. ELAV-GAL4>UAS-mACR-A-RNAi-2, UAS-DCR | < 0.01 | ** |
|  |  | Dunnett’s T3 Post-hoc multiple comparisons test | 1. ELAV-GAL4 2. UAS-mACR-A-RNAi-2, UAS-DCR | 0.6584 | n.s. |
|  |  |  | 1. ELAV-GAL4 2. ELAV-GAL4>UAS-mACR-A-RNAi-2, UAS-DCR | 0.0214 | * |
|  |  |  | 1. UAS-mACR-A-RNAi-2, UAS-DCR 2. ELAV-GAL4>UAS-mACR-A-RNAi-2, UAS-DCR | 0.0178 | * |
| Figure 1C | Learning index  MCH | One way non parametric ANOVA -  Kruskal-Wallis test | ANOVA   1. OK107-GAL4 2. UAS-mAChR-A-RNAi 2, UAS-DCR 3. OK107-GAL4>UAS-mAChR-A-RNAi 2, UAS-DCR | 0.0029 | ** |
|  |  | Dunn's multiple comparisons test | 1. OK107-GAL4 2. UAS-mAChR-A-RNAi 2, UAS-DCR | >0.9999 | n.s. |
|  |  |  | 1. OK107-GAL4 2. OK107-GAL4>UAS-mAChR-A-RNAi 2, UAS-DCR | 0.0203 | * |
|  |  |  | 1. UAS-mAChR-A-RNAi 2, UAS-DCR 2. OK107-GAL4>UAS-mAChR-A-RNAi 2, UAS-DCR | 0.0048 | ** |
| Figure 1C | Learning index  MCH | One way non parametric ANOVA -  Kruskal-Wallis test | ANOVA   1. OK107-GAL4 2. UAS-mAChR-A-RNAi 1 3. OK107-GAL4>UAS-mAChR-A-RNAi 1 | 0.0004 | *** |
|  |  | Dunn's multiple comparisons test | 1. OK107-GAL4 2. UAS-mAChR-A-RNAi 1 | >0.9999 | n.s. |
|  |  |  | 1. OK107-GAL4 2. OK107-GAL4>UAS-mAChR-A-RNAi 1 | 0.0204 | * |
|  |  |  | 1. UAS-mAChR-A-RNAi 1 2. OK107-GAL4>UAS-mAChR-A-RNAi 1 | 0.0003 | *** |
| Figure 1C | Learning index  OCT | One way non parametric ANOVA -  Kruskal-Wallis test | ANOVA   1. OK107-GAL4 2. UAS-mAChR-A-RNAi 2, UAS-DCR 3. OK107-GAL4>UAS-mAChR-A-RNAi 2, UAS-DCR | 0.0069 | ** |
|  |  | Dunn's multiple comparisons test | 1. OK107-GAL4 2. UAS-mAChR-A-RNAi 2, UAS-DCR | >0.9999 | n.s. |
|  |  |  | 1. OK107-GAL4 2. OK107-GAL4>UAS-mAChR-A-RNAi 2, UAS-DCR | 0.0168 | * |
|  |  |  | 1. UAS-mAChR-A-RNAi 2, UAS-DCR 2. OK107-GAL4>UAS-mAChR-A-RNAi 2, UAS-DCR | 0.0210 | * |
| Figure 1C | Learning index  OCT | One way non parametric ANOVA -  Kruskal-Wallis test | ANOVA   1. OK107-GAL4 2. UAS-mAChR-A-RNAi 1 3. OK107-GAL4>UAS-mAChR-A-RNAi 1 | 0.0077 | ** |
|  |  | Dunn's multiple comparisons test | 1. OK107-GAL4 2. UAS-mAChR-A-RNAi 1 | >0.9999 | n.s. |
|  |  |  | 1. OK107-GAL4 2. OK107-GAL4>UAS-mAChR-A-RNAi 1 | 0.0363 | * |
|  |  |  | 1. UAS-mAChR-A-RNAi 1 2. OK107-GAL4>UAS-mAChR-A-RNAi 1 | 0.0145 | * |
| Figure 1D | Naïve avoidance  OCT | One way non parametric ANOVA -  Kruskal-Wallis test | ANOVA   1. OK107-GAL4 2. UAS-mAChR-A-RNAi 1 3. OK107-GAL4>UAS-mAChR-A-RNAi 1 | 0.4197 | n.s. |
|  |  | Dunn's multiple comparisons test | 1. OK107-GAL4 2. UAS-mAChR-A-RNAi 1 | >0.6119 | n.s. |
|  |  |  | 1. OK107-GAL4 2. OK107-GAL4>UAS-mAChR-A-RNAi 1 | >0.9999 | n.s. |
|  |  |  | 1. UAS-mAChR-A-RNAi 1 2. OK107-GAL4>UAS-mAChR-A-RNAi 1 | >0.9999 | n.s. |
| Figure 1D | Naïve avoidance  MCH | One way non parametric ANOVA -  Kruskal-Wallis test | ANOVA   1. OK107-GAL4 2. UAS-mAChR-A-RNAi 1 3. OK107-GAL4>UAS-mAChR-A-RNAi 1 | 0.1517 | n.s. |
|  |  | Dunn's multiple comparisons test | 1. OK107-GAL4 2. UAS-mAChR-A-RNAi 1 | >0.5945 | n.s. |
|  |  |  | 1. OK107-GAL4 2. OK107-GAL4>UAS-mAChR-A-RNAi 1 | >0.1634 | n.s. |
|  |  |  | 1. UAS-mAChR-A-RNAi 1 2. OK107-GAL4>UAS-mAChR-A-RNAi 1 | >0.9999 | n.s. |
| Figure 1E | Learning with Gal80ts | One way non parametric ANOVA -  Kruskal-Wallis test | ANOVA   1. tubP-GAL80ts, OK107-GAL4 2. UAS-mAChR-A-RNAi 1 3. tubP-GAL80ts, OK107-GAL4>UAS-mAChR-A-RNAi 1 31°C 4. tubP-GAL80ts, OK107-GAL4>UAS-mAChR-A-RNAi 1 23°C | < 0.0001 | **** |
|  |  | Dunn's multiple comparisons test | 1. tubP-GAL80ts, OK107-GAL4 2. UAS-mAChR-A-RNAi 1 | >0.9999 | n.s. |
|  |  |  | 1. tubP-GAL80ts, OK107-GAL4 2. tubP-GAL80ts, OK107-GAL4>UAS-mAChR-A-RNAi 1 31°C | 0.0012 | ** |
|  |  |  | 1. tubP-GAL80ts, OK107-GAL4 2. tubP-GAL80ts, OK107-GAL4>UAS-mAChR-A-RNAi 1 23°C | >0.9999 | n.s. |
|  |  |  | 1. UAS-mAChR-A-RNAi 1 2. tubP-GAL80ts, OK107-GAL4>UAS-mAChR-A-RNAi 1 31°C | < 0.0001 | **** |
|  |  |  | 1. UAS-mAChR-A-RNAi 1 2. tubP-GAL80ts, OK107-GAL4>UAS-mAChR-A-RNAi 1 23°C | >0.6606 | n.s. |
|  |  |  | 1. tubP-GAL80ts, OK107-GAL4>UAS-mAChR-A-RNAi 1 31°C 2. tubP-GAL80ts, OK107-GAL4>UAS-mAChR-A-RNAi 1 23°C | 0.0154 | * |
| Figure 2B |  | One way non parametric ANOVA -  Kruskal-Wallis test | ANOVA   1. MB247-GAL4 2. UAS-mAChR-A-RNAi 1 3. MB247-GAL4>UAS-mAChR-A-RNAi 1 | < 0.0001 | **** |
|  |  | Dunn's multiple comparisons test | 1. MB247-GAL4 2. UAS-mAChR-A-RNAi 1 | >0.9999 | n.s. |
|  |  |  | 1. MB247-GAL4 2. MB247-GAL4>UAS-mAChR-A-RNAi 1 | 0.0016 | ** |
|  |  |  | 1. UAS-mAChR-A-RNAi 1 2. MB247-GAL4>UAS-mAChR-A-RNAi 1 | 0.0007 | *** |
|  |  | One way non parametric ANOVA -  Kruskal-Wallis test | ANOVA   1. c305a-GAL4 2. UAS-mAChR-A-RNAi 1 3. c305a-GAL4>UAS-mAChR-A-RNAi 1 | 0.4232 | n.s. |
|  |  | Dunn's multiple comparisons test | 1. c305a-GAL4 2. UAS-mAChR-A-RNAi 1 | >0.6742 | n.s. |
|  |  |  | 1. c305a-GAL4 2. OK107-GAL4>UAS-mAChR-A-RNAi 1 | >0.9999 | n.s. |
|  |  |  | 1. UAS-mAChR-A-RNAi 1 2. c305a-GAL4>UAS-mAChR-A-RNAi 1 | >0.8844 | n.s. |
|  |  | One way non parametric ANOVA -  Kruskal-Wallis test | ANOVA   1. UAS-mAChR-A-RNAi 1 2. R45H04-lexA>LexAop-GAL80, MB247-GAL4 3. R45H04-lexA>LexAop-GAL80, MB247-GAL4>UAS-mAChR-A-RNAi 1 | 0.1061 | n.s. |
|  |  | Dunn's multiple comparisons test | 1. UAS-mAChR-A-RNAi 1 2. R45H04-lexA>LexAop-GAL80, MB247-GAL4 | 0.1871 | n.s. |
|  |  |  | 1. UAS-mAChR-A-RNAi 1 2. R45H04-lexA>LexAop-GAL80, MB247-GAL4>UAS-mAChR-A-RNAi 1 | 0.2150 | n.s. |
|  |  |  | 1. R45H04-lexA>LexAop-GAL80, MB247-GAL4 2. R45H04-lexA>LexAop-GAL80, MB247-GAL4>UAS-mAChR-A-RNAi 1 | >0.9999 | n.s. |
|  |  | One way non parametric ANOVA -  Kruskal-Wallis test | ANOVA   1. UAS-mAChR-A-RNAi 1 2. R44E04-lexA>LexAop-GAL80, MB247-GAL4, 3. R44E04-lexA>LexAop-GAL80, MB247-GAL4>UAS-mAChR-A-RNAi 1 | <0.0001 | **** |
|  |  | Dunn's multiple comparisons test | 1. UAS-mAChR-A-RNAi 1 2. R44E04-lexA>LexAop-GAL80, MB247-GAL4, | >0.9999 | n.s. |
|  |  |  | 1. UAS-mAChR-A-RNAi 1 2. R44E04-lexA>LexAop-GAL80, MB247-GAL4>UAS-mAChR-A-RNAi 1 | <0.0001 | **** |
|  |  |  | 1. R44E04-lexA>LexAop-GAL80, MB247-GAL4, 2. R44E04-lexA>LexAop-GAL80, MB247-GAL4>UAS-mAChR-A-RNAi 1 | 0.0003 | *** |
| Figure 3B | KC responses to MCH with or without mAChR-A-RNAi | 2-way ANOVA | Main effect of genotype (OK107-GAL4, UAS-dcr2 vs. OK107-GAL4, UAS-dcr2, UAS-mAChR-A-RNAi 2) | < 0.0001 | **** |
|  |  |  | Main effect of lobe ( α, α’, β, β’, γ, calyx) | 0.0009 | *** |
|  |  |  | Interaction between genotype and lobe | 0.7096 | n.s. |
|  |  | Holm-Sidak multiple comparison test | dcr2 alone vs. mCAhR-A-RNAi 2, α‘ lobe | 0.0717 | n.s. |
|  |  |  | dcr2 alone vs. mCAhR-A-RNAi 2, β‘ lobe | 0.0319 | n.s. |
|  |  |  | dcr2 alone vs. mCAhR-A-RNAi 2, α lobe | 0.016 | * |
|  |  |  | dcr2 alone vs. mCAhR-A-RNAi 2, β lobe | 0.0717 | n.s. |
|  |  |  | dcr2 alone vs. mCAhR-A-RNAi 2, γ lobe | 0.0003 | *** |
|  |  |  | dcr2 alone vs. mCAhR-A-RNAi 2, calyx | 0.0196 | * |
|  | KC responses to OCT with or without mAChR-A-RNAi | 2-way ANOVA | Main effect of genotype (OK107-GAL4, UAS-dcr2 vs. OK107-GAL4, UAS-dcr2, UAS-mAChR-A-RNAi 2) | <0.0001 | **** |
|  |  |  | Main effect of lobe ( α, α’, β, β’, γ, calyx) | <0.0001 | **** |
|  |  |  | Interaction between genotype and lobe | 0.0086 | ** |
|  |  | Holm-Sidak multiple comparison test | dcr2 alone vs. mCAhR-A-RNAi 2, α‘ lobe | 0.6018 | n.s. |
|  |  |  | dcr2 alone vs. mCAhR-A-RNAi 2, β‘ lobe | 0.6018 | n.s. |
|  |  |  | dcr2 alone vs. mCAhR-A-RNAi 2, α lobe | 0.5748 | n.s. |
|  |  |  | dcr2 alone vs. mCAhR-A-RNAi 2, β lobe | 0.1481 | n.s. |
|  |  |  | dcr2 alone vs. mCAhR-A-RNAi 2, γ lobe | 0.0007 | *** |
|  |  |  | dcr2 alone vs. mCAhR-A-RNAi 2, calyx | <0.0001 | **** |
| Figure 4B | Sparseness | 2-way repeated measures ANOVA (paired across odors) | Main effect of genotype (dcr2 alone vs. mAChR-A-RNAi2) | 0.3811 | n.s. |
|  |  |  | Main effect of odor (MCH vs OCT) | 0.0001 | *** |
|  |  |  | Interaction between genotype and odor | 0.9954 | n.s. |
|  |  | Holm-Sidak multiple comparison test | dcr2 alone vs. mAChR-A-RNAi 2, MCH | 0.691 | n.s |
|  |  |  | dcr2 alone vs. mAChR-A-RNAi 2, OCT | 0.691 | n.s. |
| Figure 4C | Correlation | Unpaired t-test | dcr2 alone vs. mAChR-A-RNAi 2 | 0.7547 | n.s. |
| Figure 4F | γ-only driver, KC responses with or without mAChR-A-RNAi - in gamma lobe and calyx, with MCH or OCT | 2-way repeated measures ANOVA | Main effect of genotype | 0.0004 | *** |
|  |  | Holm-Sidak multiple comparison test | control vs. mAChR-A-RNAi 1, MCH, calyx | 0.0304 | * |
|  |  |  | control vs. mAChR-A-RNAi 1, OCT, calyx | 0.0013 | ** |
|  |  |  | control vs. mAChR-A-RNAi 1, MCH, gamma lobe | 0.8083 | n.s. |
|  |  |  | control vs. mAChR-A-RNAi 1, OCT, gamma lobe | 0.0262 | * |
| Figure 4H | Sparseness, γ KCs only | 2-way repeated measures ANOVA (paired across odors) | Main effect of genotype (control vs. RNAi) | 0.7693 | n.s. |
|  |  |  | Main effect of odor (MCH vs OCT) | 0.3838 | n.s. |
|  |  |  | Interaction between genotype and odor | 0.2621 | n.s. |
|  |  | Holm-Sidak multiple comparison test | control vs. RNAi, MCH | 0.8908 | n.s. |
|  |  |  | control vs. RNAi, OCT | 0.3261 | n.s. |
| Figure 4I | Correlation | Unpaired Welch-corrected t-test | control vs. RNAi | 0.3249 | n.s. |
| Figure 5B | KC responses to MCH before and after bath muscarine (Max. ∆F/F) | 2-way repeated measures ANOVA  (matching across drug treatment and across lobes)  n = 11 hemispheres (6 flies) | Main effect of muscarine treatment | <0.0001 | **** |
|  |  |  | Main effect of lobe ( α, α’, β, β’, γ, calyx) | 0.5052 | n.s. |
|  |  |  | Interaction between muscarine and lobe | 0.0166 | * |
|  |  | Holm-Sidak multiple comparison test | before vs. +muscarine, calyx | 0.0205 | * |
|  |  |  | before vs. +muscarine, α‘ lobe | <0.0001 | **** |
|  |  |  | before vs. +muscarine, β‘ lobe | <0.0001 | **** |
|  |  |  | before vs. +muscarine, α lobe | <0.0001 | **** |
|  |  |  | before vs. +muscarine, β lobe | 0.0011 | ** |
|  |  |  | before vs. +muscarine, γ lobe | <0.0001 | **** |
|  | KC responses to OCT before and after bath muscarine (Max. ∆F/F) | 2-way repeated measures ANOVA  (matching across drug treatment and across lobes)  n = 11 hemispheres (6 flies) | Main effect of muscarine treatment | 0.0007 | *** |
|  |  |  | Main effect of lobe ( α, α’, β, β’, γ, calyx) | 0.0448 | * |
|  |  |  | Interaction between muscarine and lobe | 0.582 | n.s. |
|  |  | Holm-Sidak multiple comparison test | before vs. +muscarine, calyx | 0.0002 | *** |
|  |  |  | before vs. +muscarine, α‘ lobe | <0.0001 | **** |
|  |  |  | before vs. +muscarine, β‘ lobe | <0.0001 | **** |
|  |  |  | before vs. +muscarine, α lobe | <0.0001 | *** |
|  |  |  | before vs. +muscarine, β lobe | <0.0001 | **** |
|  |  |  | before vs. +muscarine, γ lobe | 0.0012 | ** |
| Figure 5C | PN responses in calyx | 2-way repeated measures ANOVA (matching across drug treatment and odor) | Main effect of odor | 0.8587 | n.s. |
|  |  |  | Main effect of muscarine treatment | 0.4955 | n.s. |
|  |  |  | Interaction between odor and treatment | 0.2699 | n.s. |
|  |  | Individual paired t-test for MCH (compare to power analysis below) |  | 0.1951 | n.s. |
|  |  | Individual paired t-test for OCT (compare to power analysis below) |  | 0.7406 | n.s. |
|  |  | Power analysis | The effect size of muscarine treatment on the γ lobe’s MCH response is 2.02. n = 5 gives 97% chance of detecting such a large effect with a paired t-test |  |  |
| Figure 5D | KC responses with muscarine, APL unlabeled control - MCH | 2-way repeated measures ANOVA (matching across drug treatment and lobe | Main effect of muscarine treatment | 0.0024 | ** |
|  |  |  | Main effect of lobe ( α, α’, β, β’, γ, calyx) | 0.1006 | n.s. |
|  |  |  | Interaction between muscarine and lobe | 0.4808 | n.s. |
|  |  | Holm-Sidak multiple comparison test | before vs. +muscarine, calyx | <0.0001 | **** |
|  |  |  | before vs. +muscarine, α‘ lobe | 0.0039 | ** |
|  |  |  | before vs. +muscarine, β‘ lobe | 0.0007 | *** |
|  |  |  | before vs. +muscarine, α lobe | <0.0001 | **** |
|  |  |  | before vs. +muscarine, β lobe | 0.0002 | *** |
|  |  |  | before vs. +muscarine, γ lobe | 0.0002 | *** |
|  | KC responses with muscarine, APL unlabeled control - OCT | 2-way repeated measures ANOVA (matching across drug treatment and lobe | Main effect of muscarine treatment | 0.0564 | n.s. |
|  |  |  | Main effect of lobe ( α, α’, β, β’, γ, calyx) | 0.0107 | n.s. |
|  |  |  | Interaction between muscarine and lobe | 0.2393 | n.s. |
|  |  | Holm-Sidak multiple comparison test | before vs. +muscarine, calyx | <0.0001 | **** |
|  |  |  | before vs. +muscarine, α‘ lobe | 0.0006 | *** |
|  |  |  | before vs. +muscarine, β‘ lobe | 0.0057 | ** |
|  |  |  | before vs. +muscarine, α lobe | <0.0001 | **** |
|  |  |  | before vs. +muscarine, β lobe | <0.0001 | **** |
|  |  |  | before vs. +muscarine, γ lobe | 0.0009 | *** |
|  | KC responses with muscarine, APL>TNT - MCH | 2-way repeated measures ANOVA (matching across drug treatment and lobe | Main effect of muscarine treatment | 0.0023 | ** |
|  |  |  | Main effect of lobe ( α, α’, β, β’, γ, calyx) | 0.0156 | * |
|  |  |  | Interaction between muscarine and lobe | 0.0908 | n.s. |
|  |  | Holm-Sidak multiple comparison test | before vs. +muscarine, calyx | 0.0107 | * |
|  |  |  | before vs. +muscarine, α‘ lobe | <0.0001 | **** |
|  |  |  | before vs. +muscarine, β‘ lobe | <0.0001 | **** |
|  |  |  | before vs. +muscarine, α lobe | 0.0035 | ** |
|  |  |  | before vs. +muscarine, β lobe | 0.0035 | ** |
|  |  |  | before vs. +muscarine, γ lobe | 0.0031 | ** |
|  | KC responses with muscarine, APL>TNT - OCT | 2-way repeated measures ANOVA (matching across drug treatment and lobe | Main effect of muscarine treatment | <0.0001 | **** |
|  |  |  | Main effect of lobe ( α, α’, β, β’, γ, calyx) | 0.0001 | *** |
|  |  |  | Interaction between muscarine and lobe | 0.0128 | * |
|  |  | Holm-Sidak multiple comparison test | before vs. +muscarine, calyx | 0.0002 | *** |
|  |  |  | before vs. +muscarine, α‘ lobe | <0.0001 | **** |
|  |  |  | before vs. +muscarine, β‘ lobe | <0.0001 | **** |
|  |  |  | before vs. +muscarine, α lobe | <0.0001 | **** |
|  |  |  | before vs. +muscarine, β lobe | <0.0001 | **** |
|  |  |  | before vs. +muscarine, γ lobe | 0.0006 | *** |
| Figure 5E | (KC response after muscarine) / (KC response before muscarine), MCH | 2-way repeated measures ANOVA (matching across lobe) | Main effect of tetanus toxin expression (APL unlabeled vs. APL>TNT) | 0.1541 | n.s. |
|  |  |  | Main effect of lobe ( α, α’, β, β’, γ, calyx) | 0.3694 | n.s. |
|  |  |  | Interaction between lobe and tetanus toxin expression | 0.0229 | * |
|  |  | Holm-Sidak multiple comparison test | APL unlabeled vs. APL>TNT, calyx | 0.2367 | n.s. |
|  |  |  | APL unlabeled vs. APL>TNT, α‘ lobe | 0.5099 | n.s. |
|  |  |  | APL unlabeled vs. APL>TNT, β‘ lobe | 0.8107 | n.s. |
|  |  |  | APL unlabeled vs. APL>TNT, α lobe | 0.3311 | n.s. |
|  |  |  | APL unlabeled vs. APL>TNT, β lobe | 0.8107 | n.s. |
|  |  |  | APL unlabeled vs. APL>TNT, γ lobe | 0.0532 | n.s. |
|  | (KC response after muscarine) / (KC response before muscarine), OCT | 2-way repeated measures ANOVA (matching across lobe) | Main effect of tetanus toxin expression (APL unlabeled vs. APL>TNT) | 0.5607 | n.s. |
|  |  |  | Main effect of lobe ( α, α’, β, β’, γ, calyx) | 0.3210 | n.s. |
|  |  |  | Interaction between lobe and tetanus toxin expression | 0.1278 | n.s. |
|  |  | Holm-Sidak multiple comparison test | APL unlabeled vs. APL>TNT, calyx | 0.9787 | n.s. |
|  |  |  | APL unlabeled vs. APL>TNT, α‘ lobe | 0.8786 | n.s. |
|  |  |  | APL unlabeled vs. APL>TNT, β‘ lobe | 0.8786 | n.s. |
|  |  |  | APL unlabeled vs. APL>TNT, α lobe | 0.8786 | n.s. |
|  |  |  | APL unlabeled vs. APL>TNT, β lobe | 0.8786 | n.s. |
|  |  |  | APL unlabeled vs. APL>TNT, γ lobe | 0.8786 | n.s. |
| Figure 6C | Picospritzing muscarine on calyx, mean ∆F/F 0 – 1 s after application | One-sample t test - hypothetical value 0 - Bonferroni correction | Muscarine response calyx | 0.039 | * |
|  |  |  | Muscarine response α‘ lobe | >0.99 | n.s. |
|  |  |  | Muscarine response β‘ lobe | >0.99 | n.s. |
|  |  |  | Muscarine response α lobe | 0.027 | * |
|  |  |  | Muscarine response β lobe | >0.99 | n.s. |
|  |  |  | Muscarine response γ lobe | 0.576 | n.s. |
| Figure 6E | KC responses to MCH before and after muscarine puff on calyx on opposite side | 2-way repeated measures ANOVA (matching across drug treatment and lobe | Main effect of muscarine treatment | 0.3349 | n.s. |
|  |  |  | Main effect of lobe ( α, α’, β, β’, γ, calyx) | 0.1905 | n.s. |
|  |  |  | Interaction between muscarine and lobe | 0.2388 | n.s. |
|  |  | Holm-Sidak multiple comparisons test | before vs. +muscarine, calyx | 0.8860 | n.s. |
|  |  |  | before vs. +muscarine, α‘ lobe | 0.9441 | n.s. |
|  |  |  | before vs. +muscarine, β‘ lobe | 0.9441 | n.s. |
|  |  |  | before vs. +muscarine, α lobe | 0.9441 | n.s. |
|  |  |  | before vs. +muscarine, β lobe | 0.0158 | * |
|  |  |  | before vs. +muscarine, γ lobe | 0.7936 | n.s. |
|  | KC responses to OCT before and after muscarine puff on calyx on opposite side | 2-way repeated measures ANOVA (matching across drug treatment and lobe | Main effect of muscarine treatment | 0.3984 | n.s. |
|  |  |  | Main effect of lobe ( α, α’, β, β’, γ, calyx) | 0.3595 | n.s. |
|  |  |  | Interaction between muscarine and lobe | 0.3699 | n.s. |
|  |  | Holm-Sidak multiple comparisons test | before vs. +muscarine, calyx | 0.9403 | n.s. |
|  |  |  | before vs. +muscarine, α‘ lobe | 0.9313 | n.s. |
|  |  |  | before vs. +muscarine, β‘ lobe | 0.8593 | n.s. |
|  |  |  | before vs. +muscarine, α lobe | 0.3680 | n.s. |
|  |  |  | before vs. +muscarine, β lobe | 0.5733 | n.s. |
|  |  |  | before vs. +muscarine, γ lobe | 0.8593 | n.s. |
|  | KC responses to MCH before and after muscarine puff on calyx on same side | 2-way repeated measures ANOVA (matching across drug treatment and lobe | Main effect of muscarine treatment | 0.0227 | * |
|  |  |  | Main effect of lobe ( α, α’, β, β’, γ, calyx) | 0.1291 | n.s. |
|  |  |  | Interaction between muscarine and lobe | 0.2751 | n.s. |
|  |  | Holm-Sidak multiple comparisons test | before vs. +muscarine, calyx | 0.5295 | n.s. |
|  |  |  | before vs. +muscarine, α‘ lobe | 0.0048 | ** |
|  |  |  | before vs. +muscarine, β‘ lobe | 0.0624 | n.s. |
|  |  |  | before vs. +muscarine, α lobe | 0.5295 | n.s. |
|  |  |  | before vs. +muscarine, β lobe | 0.0400 | * |
|  |  |  | before vs. +muscarine, γ lobe | 0.2561 | n.s. |
|  | KC responses to OCT before and after muscarine puff on calyx on same side | 2-way repeated measures ANOVA (matching across drug treatment and lobe | Main effect of muscarine treatment | 0.0004 | *** |
|  |  |  | Main effect of lobe ( α, α’, β, β’, γ, calyx) | 0.0034 | ** |
|  |  |  | Interaction between muscarine and lobe | <0.0001 | **** |
|  |  | Holm-Sidak multiple comparisons test | before vs. +muscarine, calyx | 0.2421 | n.s. |
|  |  |  | before vs. +muscarine, α‘ lobe | <0.0001 | **** |
|  |  |  | before vs. +muscarine, β‘ lobe | <0.0001 | **** |
|  |  |  | before vs. +muscarine, α lobe | 0.2421 | n.s. |
|  |  |  | before vs. +muscarine, β lobe | 0.0005 | *** |
|  |  |  | before vs. +muscarine, γ lobe | 0.0075 | ** |
| Figure 7B | Real time PCR | Welch ANOVA | ANOVA   1. W^1118^ 2. MiMIC mAChR-A-Stop 3. MiMIC mAChR-A-GAL4 | 0.0001 | *** |
|  |  | Dunnett’s T3 Post-hoc multiple comparisons test | 1. W^1118^ 2. MiMIC mAChR-A-Stop | 0.0064 | ** |
|  |  |  | 1. W^1118^ 2. MiMIC mAChR-A-GAL4 | 0.0484 | * |
|  |  |  | 1. MiMIC mAChR-A-Stop 2. MiMIC mAChR-A-GAL4 | 0.0011 | ** |
| Figure 7C | Behavior | One way non parametric ANOVA -  Kruskal-Wallis test | 1. MiMIC mAChR-A-Stop 2. MiMIC mAChR-A-Stop;MB247-GAL4 3. MiMIC mAChR-A-Stop;UAS-mAChR-A 4. MiMIC mAChR-A-Stop; MB247-GAL4>UAS-mAChR-A | <0.01 | ** |
|  |  | Dunn's multiple comparisons test | 1. MiMIC mAChR-A-Stop 2. MiMIC mAChR-A-Stop;MB247-GAL4 | >0.9999 | n.s. |
|  |  |  | 1. MiMIC mAChR-A-Stop 2. MiMIC mAChR-A-Stop;UAS-mAChR-A | >0.9999 | n.s. |
|  |  |  | 1. MiMIC mAChR-A-Stop 2. MiMIC mAChR-A-Stop; MB247-GAL4>UAS-mAChR-A | 0.0306 | * |
|  |  |  | 1. MiMIC mAChR-A-Stop;MB247-GAL4 2. MiMIC mAChR-A-Stop;UAS-mAChR-A | >0.9999 | n.s. |
|  |  |  | 1. MiMIC mAChR-A-Stop;MB247-GAL4 2. MiMIC mAChR-A-Stop; MB247-GAL4>UAS-mAChR-A | 0.0180 | * |
|  |  |  | 1. MiMIC mAChR-A-Stop;UAS-mAChR-A 2. MiMIC mAChR-A-Stop; MB247-GAL4>UAS-mAChR-A | 0.0020 | ** |
| Figure 8B | MBON odor responses | Mann Whitney test | Ratio between OCT and MCH odor responses.   1. OK107, R12G04>GCaMP - mock training 2. OK107, R12G04>GCaMP - training | 0.0159 | * |
|  |  | Mann Whitney test | Ratio between OCT and MCH odor responses.   1. OK107>DM type A RNAi, R12G04>GCaMP - mock training 2. OK107>DM type A RNAi, R12G04>GCaMP - training | 0.1775 | n.s. |
| Figure 1—figure supplement 1, panel A | Behavior  No shock | One sample t-test | 1. OK107-GAL4 | 0.3900 | n.s. |
|  |  |  | 1. UAS-mAChR-A-RNAi 1 | 0.3595 | n.s. |
|  |  |  | 1. OK107-GAL4>UAS-mAChR-A-RNAi 1 | 0.3305 | n.s. |
|  | Behavior  90V | One way non parametric ANOVA -  Kruskal-Wallis test | ANOVA   1. OK107-GAL4 2. UAS-mAChR-A-RNAi 1   OK107-GAL4>UAS-mAChR-A-RNAi 1 | 0.1309 | n.s. |
|  |  | Dunn's multiple comparisons test | 1. OK107-GAL4   UAS-mAChR-A-RNAi 1 | 0.3904 | n.s. |
|  |  |  | 1. OK107-GAL4   OK107-GAL4>UAS-mAChR-A-RNAi 1 | >0.9999 | n.s. |
|  |  |  | 1. UAS-mAChR-A-RNAi 1   OK107-GAL4>UAS-mAChR-A-RNAi 1 | 0.1605 | n.s. |
| Figure 1—figure supplement 1, panel B | Behavior | Mann Whitney test  Bonferroni multiple comparison | 1. OK107-GAL4 | 0.0246 | * |
|  |  |  | 1. UAS-mAChR-A-RNAi 1 | <0.0001 | **** |
|  |  |  | 1. OK107-GAL4>UAS-mAChR-A-RNAi 1 | 0.0018 | ** |
|  |  | 2-way ANOVA | Main effect of genotype   1. OK107-GAL4 2. UAS-mAChR-A-RNAi 1 3. OK107-GAL4, UAS-mAChR-A-RNAi 1 | 0.2687 | n.s. |
|  |  |  | Main effect of shock   1. Shock 2. Mock training | <0.0001 | **** |
|  |  |  | Interaction   1. OK107-GAL4, shock 2. OK107-GAL4, mock training 3. UAS-mAChR-A-RNAi 1, shock 4. UAS-mAChR-A-RNAi 1, mock training 5. OK107-GAL4, UAS-mAChR-A-RNAi 1, shock   OK107-GAL4, UAS-mAChR-A-RNAi 1, mock training | < 0.0001 | **** |
|  |  | 2-way ANOVA | Main effect of genotype   1. OK107-GAL4 2. OK107-GAL4, UAS-mAChR-A-RNAi 1 | 0.1052 | n.s. |
|  |  |  | Main effect of shock   1. Shock 2. Mock training | <0.0001 | **** |
|  |  |  | Interaction   1. OK107-GAL4, shock 2. OK107-GAL4, mock training 3. OK107-GAL4, UAS-mAChR-A-RNAi 1, shock   OK107-GAL4, UAS-mAChR-A-RNAi 1, mock training | 0.4352 | n.s. |
