# Supplementary material for: Inhibitory muscarinic acetylcholine receptors enhance aversive olfactory learning in adult Drosophila
Source: eLife. 2019 Jun 19;8:e48264. doi: 10.7554/eLife.48264 (PMC6641838; doi:10.7554/eLife.48264)
Supplement: Supplementary file 2. [file elife-48264-supp2.docx]

**Table S2: Detailed genotypes used in this study**

| **Figure** | **Shorthand** | **Full genotype** |
| --- | --- | --- |
| 1A, 1–figure supplement 1 | N/A | *elav-GAL4/+* or *Y*  *UAS-mAChR-A-RNAi 1/+*  *elav-GAL4/+* or *Y; +; UAS-mAChR-A-RNAi 1/+*  *UAS-dcr2/+; UAS-mAChR-A-RNAi 2/+*  *elav-GAL4/+* or *Y; UAS-dcr2/+; UAS-mAChR-A-RNAi 2/+* |
| 1B,C | N/A | *OK107-GAL4/+*  *UAS-mAChR-A-RNAi 1/+*  *UAS-mAChR-A-RNAi 1/+; +; OK107-GAL4/+*  *UAS-dcr2/+; UAS-mAChR-A-RNAi 2/+*  *UAS-dcr2/+; UAS-mAChR-A-RNAi 2/+; OK107-GAL4/+* |
| 1E | N/A | *tub-GAL80^ts^/+; +; OK107-GAL4/+*  *UAS-mAChR-A-RNAi 1/+*  *tub-GAL80^ts^/+; UAS-mAChR-A-RNAi 1/+; OK107-GAL4/+* |
| 2A | N/A | *MiMIC-mAChR-A-GAL4; 20xUAS-6xGFP* |
| 2B | N/A | *UAS-mAChR-A-RNAi 1/+*  *mb247-GAL4/+*  *mb247-GAL4/UAS-mAChR-A-RNAi 1*  *c305a-GAL4/+*  *c305a-GAL4/+; UAS-mAChR-A-RNAi 1/+*  *{lexAop-GAL80}su(Hw)attP5/R45H04-LexA; mb247-GAL4/+*  *{lexAop-GAL80}su(Hw)attP5)/R45H04-LexA; mb247-GAL4/UAS-mAChR-A-RNAi 1*  *{lexAop-GAL80}su(Hw)attP5)/R44E04-LexA; mb247-GAL4/+*  *{lexAop-GAL80}su(Hw)attP5/R44E04-LexA; mb247-GAL4/UAS-mAChR-A-RNAi 1* |
| 2—figure supplement 2 | OK107-GAL4>UAS-GFP | *+; UAS-mCD8-GFP/+;OK107-GAL4/+* |
|  | MB247-GAL4>UAS-GFP | *+; UAS-mCD8-GFP/mb247-GAL4; +* |
|  | c305a-GAL4>UAS-GFP | *c305a-GAL4, UAS-mCD8-GFP/CyO; Sb/TM3,Ser* |
|  | R44E04-LexA>LexAop-GFP | *{R44E04-LexA}attP40/{LexAop2-mCD8-GFP}attp40/CyO; +; +* |
|  | R45H04-LexA>LexAop-GFP | *{R45H04-LexA}attP40/{LexAop2-mCD8-GFP}attp40/CyO; +; +* |
|  | MB247-GAL4>UAS-GFP R45H04-LexA>LexAop-GAL80 | *{R45H04-LexA}attP40/UAS-mCD8-GFP; mb247-GAL4/LexAop-GAL80; +* |
|  | MB247-GAL4> UAS-GCaMP6f R44E04-LexA>LexAop-GAL80 | *{R44E04-LexA}attP40/LexAop-GAL80, UAS-GCaMP6f; mb247-GAL4/+* |
|  | R12G04-LexA>LexAop-GFP | *{R12G04-LexA}attP40/LexAop2-mCD8-GFP; +; +* |
| 3,4 | OK107-GAL4>GCaMP6f, dcr2 | *{UAS-GCaMP6f}attP40/UAS-dcr2; +; OK107-GAL4/+* |
| 3,4 | OK107-GAL4>GCaMP6f, dcr2, mAChR-A-RNAi 2 | *{UAS-GCaMP6f}attP40/UAS-dcr2; UAS-mAChR-A-RNAi 2/+; OK107-GAL4/+* |
| 4 | mb247-GAL4>GCaMP6f, R44E04-LexA>Gal80 | *{lexAop-GAL80}su(Hw)attP5, {UAS-GCaMP6f}attP40/R44E04-LexA; mb247-GAL4/+* |
| 4 | mb247-GAL4>GCaMP6f, mAChR-A-RNAi 1, R44E04-LexA>Gal80 | *{lexAop-GAL80}su(Hw)attP5, {UAS-GCaMP6f}attP40/R44E04-LexA; mb247-GAL4/UAS-mAChR-A-RNAi 1* |
| 5A,B, 6, | OK107-GAL4>GCaMP6f | *{UAS-GCaMP6f}attP40/+; +; OK107-GAL4/+* |
| 5C | GH146-GAL4>GCaMP6f | *GH146-GAL4/+; UAS-GCaMP6f/+* |
| 5D | APL unlabeled, APL>TNT | *NP2631-GAL4, GH146-FLP/tub-FRT-GAL80-FRT, UAS-mCherry, UAS-TNT; mb247-LexA, lexAop-GCaMP6f/+* |
| 7A | OK107>UAS-mAChR-A | *UAS-mAChR-A-FLAG/+; OK107-GAL4/+* |
| 7C | N/A | *MiMIC mAChR-A-Stop/MiMIC mAChR-A-Stop*  *MiMIC mAChR-A-Stop/MiMIC mAChR-A-Stop; mb247-GAL4/+*  *MiMIC mAChR-A-Stop/MiMIC mAChR-A-Stop; UAS-mAChR-A-FLAG/+*  *MiMIC mAChR-A-Stop/MiMIC mAChR-A-Stop; UAS-mAChR-A-FLAG/mb247-GAL4* |
| 8 | OK107-GAL4, R12G04-LexA>GCaMP6f, mb247-dsRed | *{R12G04-LexA}attP40/{lexAop-GCaMP6f}su(Hw)attP5, mb247-dsRed; +; OK107-GAL4/+* |
| 8 | OK107-GAL4>mAChR-A-RNAi 1, R12G04-LexA>GCaMP6f, mb247-dsRed | *{R12G04-LexA}attP40/{lexAop-GCaMP6f}su(Hw)attP5, mb247-dsRed; UAS-mAChR-A-RNAi 1/+; OK107-GAL4/+* |
